# Strengthening performance-based financing as a health system approach for quality improvement

**DOI:** 10.7189/jogh.08.020305

**Published:** 2018-12

**Authors:** Wu Zeng, Daxin Sun, Dinesh Nair, Jae Eun Nam, Adrian Gheorghe

**Affiliations:** 1 Schneider Institutes for Health Policy, Brandeis University, Waltham, Massachusetts, USA; 2 School of Transportation and Civil Engineering, Fujian Agriculture and Forestry University, Fuzhou, Fujian, China; 3 The World Bank, Washington, D.C., USA; 4 Brandeis University, Waltham, Massachusetts, USA; 5 Oxford Policy Management, Oxford, UK; * Joint first authorship

Over the last decade, a significant reduction of maternal and child mortality has been achieved in low- and middle-income countries (LMICs). This is largely attributable to the substantial improvement in access to essential reproductive, maternal and child health services [1]. However, in some countries, expansion of health services has not resulted in the expected mortality reduction [2]. Low quality of care (QoC) is an important cause of this discrepancy, and it calls for putting quality improvement on the global health agenda.

As an approach to enhance QoC in LMICs, performance-based financing (PBF), which incentivizes health providers based on predetermined indicators, has been piloted or implemented in more than 30 countries. More importantly, PBF has been used as an important vehicle to catalyze health system reforms to enhance service delivery, including quality improvement (QI), in many countries.

This paper takes a system perspective to examine the current practice of PBF in strengthening health systems for QI, and provides insights for future PBF implementation. This is of particular importance in the era when countries endeavor to progress towards Universal Health Coverage (UHC) and achieve Sustainable Development Goal (SDG) 3 ensuring “healthy lives and promote well-being for all and at all ages.”

## FRAMEWORK OF THE INTERACTION AMONG PBF, HEALTH SYSTEMS, AND QUALITY OF CARE

**Figure 1:**
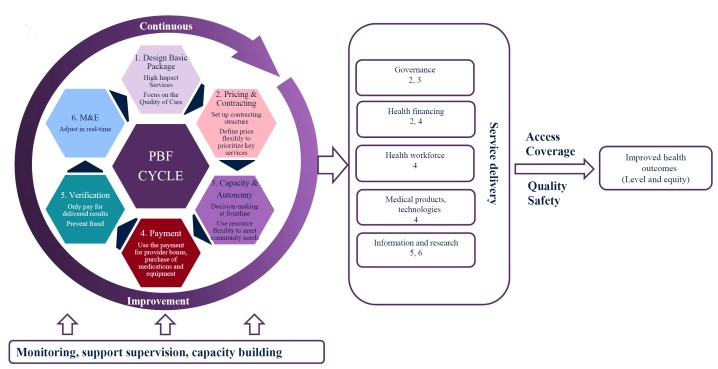
The framework of the interaction of PBF operations, health system and quality of care. PBF – performance-based financing, M&E – monitoring and evaluation.

Health systems are fundamental for ensuring good QoC and access to that care. In the WHO’s health system framework, QoC (ensuring that the care people receive is safe, effective, patient-centered, timely, efficient, and equitable) is a central component, and mediates the relationship between the building blocks of service delivery and improved health outcomes as shown on the right side of **Figure 1**. QoC and access to health care complement each other. The lack of either one would compromise the progress towards achieving better health outcomes.

PBF comprises an array of comprehensive interventions and is increasingly regarded as a health system intervention, rather than a sole contracting mechanism, to address maternal and child health (MCH) concerns by improving both the quality of and access to health care [3]. **Figure 1** shows the integration of the WHO’s health system framework and PBF’s operations that may enable PBF to influence different elements of a health care system for improving MCH, and demonstrates the multiple-dimensional impact of PBF.

## THE IMPACT OF PBF ON HEALTH SYSTEMS AND QOC

QoC is measured, to some degree, in all PBF programmes with payments to health facilities including calculation of improvements in quality. Although the main purpose of PBF is to improve the quality and quantity of health services PBF may, during implementation, trigger a series of health system changes affecting a wide range of functions of service delivery: **governance**, **financing**, **human resources, medical products/technologies**, i**nformation and research**, and thus service delivery, as shown in **Figure 1**. These are discussed briefly below.

### Governance

PBF can have significant impact on overall health governance structures and policies, which may include but are not limited to: (1) splitting purchasers from providers; (2) increasing health facility autonomy in management over human resources and medical products / technologies; (3) defining a clear role for each stakeholder of the PBF program; and (4) enhancing accountability with explicit regulations and policies on using resources. This allows health facilities to swiftly respond to their community and the populations they serve. In Cambodia, for example, PBF has resulted in strengthened operational and financial management, with strong collaboration among stakeholders [4]. Even when decentralization policies are not in place, PBF is able to mobilize resources to be used on the frontline of health service delivery at health facilities. Accompanying with the decentralization is the enhanced facility management through leadership training, regular meetings, development of strategic plans, and community engagement.

### Health financing

The core activity of PBF is to contract and financially support health providers for predefined indicators/services. It also results in a direct impact on health financing. In some countries, it is regarded as a financing approach to health providers, because the PBF program may be accompanied by a full or partial exemption of user fees charged at health facilities; and health providers may use incentive payments to compensate the loss of user fees. In Zimbabwe, the exemption of user fees has helped improve access to health facilities (personal communication with Ronald Mutasa of the World Bank).

More often, PBF is regarded as a contracting instrument for provider payment mechanisms (PPM) to achieve intended outcomes (eg, QI). Under the WHO’s advocacy, many countries have considered and started deploying strategic purchasing as one approach to enhance efficiency by switching from input-based to output-based financing. By explicitly blending or augmenting existing PPM- such as fee for service, pay by capitation, line-item budget, or global budget- PBF has the potential to hold health facilities more accountable to specific outputs. It generates room for health providers to concentrate on outputs, even under a rigid PPM such as line-item budget for public facilities.

### Human workforce

The PBF program may lead to the improvement of human resources for three major reasons: (1) part of incentive payments provided to health facilities could be used as incentive bonus to health providers, which may motivate providers to work harder; (2) the incentive could be used in a way to stimulate payment reforms within the health facility. Whoever works harder is paid better, so as to align the payment to the service delivery; (3) the autonomy granted to health facilities gives facilities the freedom to hire additional or better-qualified providers to deliver services to better meet populations needs. In Zambia, PBF contributes to recruiting and retaining health providers [5].

### Medical products and supplies

With additional resources, health facilities are able to upgrade or maintain equipment and replenish medicines to address the issue of stock out medications. In fact, the availability of essential medicines is one of the most important QoC indicators in PBF programs. Some programs also stipulate that certain percentage of incentive payments should be used for medicines and equipment. Even under the circumstance that health facilities do not have adequate incentive payments to purchase relatively costly equipment, the management at the district level could redistribute the incentive payments to meet the health facilities’ needs. All these contribute to the improved availability of products and supplies. Additionally, the strengthened information system due to PBF would allow health facilities to track the use and supply of medical products, to help the health facilities to adjust strategies accordingly. In Tanzania, an approximate eight percentage points increase in medicines and medical supplies was observed [6].

### Information and research

Furthermore, PBF cannot be implemented without enhanced information collection, particularly on incentivized indicators. To collect the necessary data or the payment, data audit and verification are routinely conducted for both implementation and evaluation purposes. The regular verification provides not only supervision supports to frontline health providers, but also mechanisms to avoid health providers’ gaming the system [3].

Although there are concerns about the lack of strong evidence regarding the impact of PBF programs on service delivery in LMICs, from a health system’s perspective, PBF programs provide an opportunity for countries to reform their health systems, with a potential to achieve greater accountability, more efficient government structure, and improved inputs (eg, medicines and supplies) for service delivery [3].

### Impact of PBF on QoC

Despite the potential of a favorable impact of PBF on the functions of a health system, a systematic review of the specific impact of PBF on quality of care was less optimistic, reporting that the only positive impact of PBF on antenatal care was primarily on structural quality [7]. The improvement in health system inputs is not necessarily translated to better QoC contributing to the reduction of maternal and child mortality, as QoC is more complex than merely enhancing health system inputs (all six building blocks except the block of service delivery). Additionally, partially due to the lack of comprehensive evaluation framework, existing evaluation of the impact of PBF generally neglects its potential impact on health system functions. This is of particular importance as it may take time to realize better quality of care.

To improve the QoC through PBF, some countries have realized that PBF should more directly target the process of service delivery, and integrate PBF with those QI initiatives that focus on process and outcome quality. Unlike many developed countries, such as the United States, that have sophisticated systems for assessing process and outcome quality, the weak information system in many LMICs hinders such development. The PBF programs in LMICs focus more on structural quality for reimbursement. A growing number of countries are considering and combining PBF with other QI programs, such as accreditation. In Afghanistan, the accreditation of primary health facilities is on the Ministry’s agenda in order to assure a basic level of quality of care and pave the way for health insurance. In Liberia, accreditation has been implemented in conjunction with an PBF program. As the indicators for accreditation in Liberia were similar to those for contracting with health facilities under PBF (including human resources, pharmacy, dispensary and storeroom, drugs and suppliers, laboratory tests, infrastructure, equipment and others) and the accreditation score was used as an indicator for contracting, it was found that the PBF program in Liberia improved accreditation scores, and accelerated the pace of health facilities to be accredited under the independent evaluation [8].

Even in countries with limited QI programs, PBF could be used as a catalyst to inspire governments to consider ways of improving QoC. In Zimbabwe, several maternal and child services, such as institutional delivery are now well utilised, however maternal and infant mortality rates continue to remain high. Results from the PBF impact evaluation propelled the Ministry of Health to focus on quality improvement as one of the key strategies to reduce maternal and child mortalities. The government of Zimbabwe and development partners have been piloting a QI program in four districts.

## MOVING FORWARD QOC UNDER PBF

In light of the limited evidence of PBF on QoC in LMICs and the potentially broad impact of PBF on health systems, policy makers ought to consider the synergy between these two elements, placing PBF as a catalyst to trigger health system changes for QI. Addressing the following three issues may help better integrate QoC under PBF programs.

### Develop a comprehensive evaluation framework that includes PBF’s impact on health systems

The key indicators used for evaluating the impact of PBF have been utilization and quality of care. As PBF is a comprehensive intervention that may affect the overall health system, it is recommended to include in the evaluation, indicators that measure the aspects of a health system, such as governance, capacity building, human resources, and medication stock-outs [9], to document pathways on how PBF could potentially affect QoC through strengthened health systems.

### Improve quality measurement indicators

The fundamental purpose of PBF is to pay for predefined services and indicators. Thus, the validity of QoC indicators plays an instrumental role in determining the success of PBF for QI. A recent review of PBF quality indicators suggests that the current quality indicators used under PBF are primarily structural, with very few process and outcome indicators [10]. This is exactly the same issue as the measurement of PBF’s impact on health systems, which primarily focused on inputs for a health system, rather than process and health outcomes for the health system. More direct process and outcome quality measures should be developed and used to determine the PBF payment.

**Figure:**
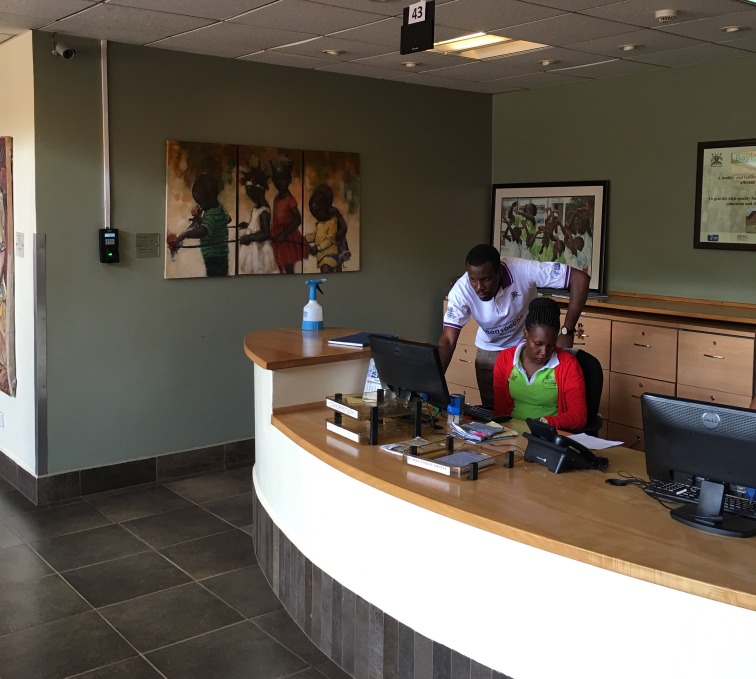
Photo: Reception at a hospital in Uganda (from the collection of Wu Zeng, used with permission)

### Integrate PBF with other QI initiatives to maximize the impact of PBF

Current measurement of QoC is a static process, where health facilities are evaluated against a set of checklists. Once health facilities meet the criteria for particular indicators, there is no incentive for them to further improve the care. Leaving QI as a static process may limit the impact of the PBF programs on QoC. QI should be treated as a dynamic process. Designing specific QI interventions (such as continuous quality improvement) that are linked to PBF ought to be considered as a more targeted intervention for QI.

## PAPERS IN THIS SPECIAL SERIES

Recognizing that QoC is essential to ensure that countries’ health systems are able to achieve intended health outcomes, this special issue is devoted to QoC under the PBF programs. Patel’s paper assembles existing evidence on evaluating QoC; Josephson’s paper highlights the need of more attention to quality measures in the checklists; Fritsche’s paper and the Kyrgyzstan case study examines innovations in measuring QoC and integrating QoC measurement with the quality improvement process.
